# Novel gateway binary vectors for rapid tripartite DNA assembly and promoter analysis with various reporters and tags in the liverwort *Marchantia polymorpha*

**DOI:** 10.1371/journal.pone.0204964

**Published:** 2018-10-04

**Authors:** Shoji Mano, Ryuichi Nishihama, Sakiko Ishida, Kazumi Hikino, Maki Kondo, Mikio Nishimura, Katsuyuki T. Yamato, Takayuki Kohchi, Tsuyoshi Nakagawa

**Affiliations:** 1 Department of Cell Biology, National Institute for Basic Biology, Okazaki, Japan; 2 Department of Basic Biology, School of Life Science, SOKENDAI (The Graduate University for Advanced Studies), Okazaki, Japan; 3 Graduate School of Biostudies, Kyoto University, Kyoto, Japan; 4 Spectrography and Bioimaging Facility, NIBB Core Research Facilities, National Institute for Basic Biology, Okazaki, Japan; 5 Faculty of Biology-Oriented Science and Technology, Kindai University, Wakayama, Japan; 6 Department of Molecular and Functional Genomics, Interdisciplinary Center for Science Research, Organization for Research, Shimane University, Matsue, Japan; CSMCRI, INDIA

## Abstract

The liverwort *Marchantia polymorpha* is an emerging model species for basal lineage plant research. In this study, two Gateway cloning-compatible binary vector series, R4pMpGWB and R4L1pMpGWB, were generated to facilitate production of transgenic *M*. *polymorpha*. The R4pMpGWB series allows tripartite recombination of any promoter and any coding sequence with a specific reporter or tag. Reporters/tags for the R4pMpGWB series are GUS, ELuc(PEST), FLAG, 3×HA, 4×Myc, mRFP1, Citrine, mCitrine, ER-targeted mCitrine and nucleus-targeted mCitrine. The R4L1pMpGWB series is suitable for promoter analysis. R4L1pMpGWB vector structure is the same as that of R4pMpGWB vectors, except that the *att*R2 site is replaced with *att*L1, enabling bipartite recombination of any promoter with a reporter or tag. Reporters/tags for the R4L1pMpGWB series are GUS, G3GFP-GUS, LUC, ELuc(PEST), Citrine, mCitrine, ER-targeted mCitrine and mCitrine-NLS. Both vector series were functional in *M*. *polymorpha* cells. These vectors will facilitate the design and assembly of plasmid constructs and generation of transgenic *M*. *polymorpha*.

## Introduction

The liverwort *Marchantia polymorpha*, a basal land plant, is an emerging model plant due to its ease of cultivation in the laboratory and amenability to genetic manipulation as well as other beneficial characteristics such as minimal gene redundancy. Recent resource improvements have facilitated the utility of *M*. *polymorpha* as a model system, such as sequence information for nuclear [1, 2], chloroplast [3] and mitochondrial [4] genomes and the availability of molecular genetic tools [5–7] and cryopreservation technologies [8]. Reliable production of transgenic *M*. *polymorpha* lines is becoming increasingly important for gene characterisation, and foreign genes have been successfully introduced into the *M*. *polymorpha* genome in several studies [5, 9–15]. Ishizaki et al. (2015) [6] developed Gateway cloning-compatible binary vectors (pMpGWB series) for the production of fusion constructs for *M*. *polymorpha*. Gateway cloning is a popular technology that allows simultaneous generation of multiple constructs containing a range of fusion genes. The pMpGWB vector series allows gene expression under the control of the Cauliflower Mosaic Virus 35S (*pro35S*) promoter or the endogenous *ELONGATION FACTOR 1α* (proMp*EF1α*) promoter as outlined previously [16]. However, tissue-specific, developmental or conditional promoters are frequently required for comprehensive analysis of gene function. In such cases, Gateway cloning-compatible binary vectors can be used to assemble desirable combinations of promoter, coding sequence and gene reporters or tags.

Previously, we developed a range of Gateway cloning-compatible vectors for use by the plant research community [17–23]. One of these Gateway cloning-compatible vectors, R4pGWB, allows coding sequences to be expressed with a reporter/tag under the regulation of any promoter [18]. However, R4pGWB vectors cannot be used for generating transgenic *M*. *polymorpha* lines, because the resistance genes used to select plants transformed with R4pGWB vectors are controlled by the promoter from *nopaline synthase* (*proNOS*) [18], which does not function in *M*. *polymorpha* [6]. The original R4pGWB vectors adopted *proNOS* to control selection in order to avoid co-suppression between coding sequence and resistance gene expression. The *proNOS* promoter can functionally drive expression of resistance genes such as *neomycin phosphotransferase II* and *hygromycin phosphotransferase* in *Arabidopsis thaliana*, but the promoter is too weak to fulfil this purpose in *M*. *polymorpha*. Ishizaki et al. (2015) [6] used the double-enhancer version of *pro35S* promoter rather than *proNOS* to drive expression of resistance genes in pMpGWB vectors. Therefore, to increase the utility of the R4pGWB vectors in *M*. *polymorpha*, we generated new vectors (R4pMpGWB series) in which *proNOS* was replaced with the double-enhancer version of *pro35S* to drive expression of resistance genes.

R4pGWB and R4pMpGWB vectors can be used to fuse a reporter/tag to a coding sequence under the control of any promoter. However, in some experiments such as promoter assays, a coding sequence is not required and promoters are positioned 5′ of the reporter sequence. Previously, we constructed R4L1pGWB vectors through modification of R4pGWB vectors [20]. In this study, new R4L1pGWB vectors for *M*. *polymorpha*, designated as R4L1pMpGWB vectors, were produced by introducing the double-enhancer version of *pro35S* to drive resistance gene expression, as in R4pMpGWB vectors.

Here, we demonstrate the utility of two novel Gateway cloning-compatible vectors for *M*. *polymorpha*: R4pMpGWB and R4L1pMpGWB. These vector series will allow rapid construction of fusion constructs regulated by the desired promoters for use in *M*. *polymorpha*.

## Materials and methods

### Plant materials and growth conditions

Transgenic and wild-type *M*. *polymorpha* plants (accessions Takaragaike-1 (Tak-1) for male and Tak-2 for female plants [5]) were grown on half-strength Gamborg’s B5 medium [24] containing 1% agar. Plants were incubated at 22°C.

### Construction of Gateway-compatible binary vectors for *M*. *polymorpha*

Primer sequences are shown in S1 Table. For all vectors, PCR-amplified DNA fragments and ligation junctions were checked by sequence analysis.

A DNA fragment corresponding to Citrine was amplified by PCR using the primers Citrine-F/Citrine-R and inserted into the Aor51HI site in pUGW1 [17] and R4L1pUGW1 [25], yielding pGWCit and R4L1pUGW07, respectively. DNA fragments corresponding to ELuc(PEST) and mCitrine, which was Arabidopsis-codon-optimized monomeric Citrine DNA fragment (kindly provided by Drs. S.S. Sugano and K. Osakabe), were amplified by PCR using the primer pairs ElucP-F/Eluc-R and AtcCit-F/AtcCit-R, and inserted into the Aor51HI site in R4pUGW1 [18] and R4L1pUGW1 [25], respectively, yielding R4pUGW31 and R4L1pUGW31 for ELuc(PEST), and R4pUGW39 and R4L1pUGW39 for mCitrine. Site-directed mutagenesis was carried out to remove an internal HindIII site in the ELuc(PEST) sequence using the primer set ElucP-sdF/ElucP-sdR.

To generate nucleus-localised mCitrine (mCit-NLS), a nucleotide sequence encoding ten amino acid residues of the nuclear localisation signal (NLS) Gln-Pro-Lys-Lys-Lys-Arg-Lys-Val-Gly-Gly was cloned to the 3′ end of the mCitrine fragment by two-step PCR amplification. First and second round PCRs were carried out using the primer sets AtcCit-F/AtcCitNLSv2-R1 and AtcCit-F/AtcCitNLSv2-R2, respectively. To generate ER-localised mCitrine (designated mCit-h), nucleotide sequences encoding the amino acid sequences Met-Ala-Arg-Leu-Thr-Ser-Ile-Ile-Ala-Leu-Phe-Ala-Val-Ala-Leu-Leu-Val-Ala-Asp-Ala-Tyr-Aal-Tyr-Arg-Thr-Met-Gly-Gly and Gly-Asp-Leu-Gly-Gly-Gly-His-His-His-His-His-His-Asp-Glu-Leu were cloned to the 5′ and 3′ ends of the mCitrine fragment, respectively, by three-step PCR amplification. First, second and third round PCRs were carried out using the primer sets mCit-hF1/mCit-hR1, mCit-hF2/mCit-hR2 and mCit-hF3/mCit-hR3, respectively. PCR-amplified DNA fragments were inserted into the Aor51HI sites in R4pUGW1 [18] and R4L1pUGW1 [25], yielding R4pUGW94 and R4L1pUGW94 for mCit-NLS, and R4pUGW95 and R4L1pUGW95 for mCit-h, respectively.

*E*. *coli* DB3.1 (Thermo Fisher Scientific, Yokohama, Japan) cultures harbouring R4pUGW and R4L1pUGW series vectors were selected on LB medium containing 50 mg/l ampicillin and 30 mg/l chloramphenicol.

Gateway vectors pMpGWB125, pMpGWB214, pMpGWB303 and pMpGWB409 [6] were digested with HindIII and SacI. Each desired DNA fragment was isolated from agarose gel, and ligated to a DNA fragment containing *attR4*—SwaI—SalI—XhoI—*Cm*^*r*^—*ccdB*—*attR2* derived from R4pUGW1 [18], yielding R4pMpGWB101, R4pMpGWB201, R4pMpGWB301 and R4pMpGWB401, respectively. R4pMpGWB101, R4pMpGWB201, R4pMpGWB301 and R4pMpGWB401 were digested with SalI and SacI, and ligated to a DNA fragment containing SalI—MluI—SacI to remove the Gateway cassette, generating intermediate plasmids R4pMpGWB101dW, R4pMpGWB201dW, R4pMpGWB301dW and R4pMpGWB401dW. pUGW11 (FLAG), pUGB14 (3×HA), pUWG17 (4×Myc), pUGW33 (GUS), pUGW54 (mRFP1, [17]) and pGWCit (Citrine) were digested with XhoI and SacI, and DNA fragments containing a reporter or a tag were inserted into SalI—SacI sites in pMpGWB101dW, R4pMpGWB201dW, R4pMpGWB301dW and R4pMpGWB401dW, generating the R4pMpGWB series containing FLAG, 3×HA, 4×Myc, GUS, mRFP1 and Citrine.

R4pUGW31, R4pUGW39, R4pUGW94 and R4pUGW95 vectors were digested with HindIII and SacI, and DNA fragments containing a reporter were inserted into HindIII—SacI sites in pMpGWB101dW, R4pMpGWB201dW, R4pMpGWB301dW and R4pMpGWB401dW, generating the R4pMpGWB series containing ELuc(PEST), mCitrine, mCit-h and mCit-NLS.

R4L1pMpGWB vectors were prepared by insertion of reporter fragments isolated from the R4L1pUGW series into pMpGWB101dW, R4pMpGWB201dW, R4pMpGWB301dW and R4pMpGWB401dW. R4L1pUGW4 (GUS, [20]), R4L1pUGW07 (Citrine), R4L1pUGW31 (ELuc(PEST)), R4L1pUGW32 (G3-GUS, [20]), R4L1pUGW35 (LUC, [20]), R4L1pUGW39, R4L1pUGW94 and R4L1pUGW95 were digested with HindIII—SacI, and DNA fragments containing a reporter or a tag were inserted into HindIII—SacI sites in pMpGWB101dW, R4pMpGWB201dW, R4pMpGWB301dW and R4pMpGWB401dW, generating the R4L1pMpGWB vector series.

*E*. *coli* DB3.1 (Thermo Fisher Scientific) cultures harbouring R4pMpGWB and R4L1pMpGWB series vectors were selected on LB medium containing 50 mg/l spectinomycin and 30 mg/l chloramphenicol.

### Construction of fusion genes

The combinations of entry clones and destination vectors used in this study are shown in S2 Table.

DNA fragments conjugated at *att*B4 and *att*B1R sites at the 5′ and 3′ ends, respectively, corresponding to the duplicated *35S* promoter in pMpGWB303, Mp*EF1α* promoter in pMpGWB303, Mp*HSP17*.*8A1* promoter in pMpGWB322 and Mp*PROTAMINE* promoter in PRMC#1, which contained the full-length *PROTAMINE* gene, were amplified by PCR using primer sets 35SpDup-FB4/35SpDup-RB1R, EF1pro-FB4/EF1pro-RB1R, MpHSP17.8A1pro-FB4/MpHSP17.8A1pro-RB1R and MpPRMproF3-B4/MpPRMproR-RB1R (S1 Table), respectively, and purified. The PCR-amplified DNA fragments were transferred to the donor vector, pDONR P4-P1R, by a BP recombination (Thermo Fisher Scientific).

DNA fragments conjugated at *att*B1 and *att*B2 sites at the 5′ and 3′ ends, respectively, corresponding to Citrine in pMpGWB306 and Lifeact-Venus (LAV) in pENTR Lifeact-Venus (kindly provided by Drs Ueda and Era [26]), were amplified by PCR using primer sets Venus-FB1/VenusPTS1-RBs and LAV-FB1/LAB-RB2 (S1 Table), respectively, and purified. The PCR-amplified DNA fragments were transferred to the donor vector, pDONR221, by a BP recombination (Thermo Fisher Scientific).

To construct *pro*Mp*EF1α*:*Citrine-PTS1* and *pro35S*:*mRFP-PTS1* fusion genes, entry clones containing cDNAs encoding Citrine-PTS1 or mRFP1-PTS1 [27] were subjected to LR recombination using the appropriate destination vectors.

To construct *pro*Mp*EF1α*:*PTS2-Citrine*, entry clones of PTS2 (encoding 48 N-terminal amino acid residues of pumpkin citrate synthase [28]) and the Mp*EF1α* promoter were subjected to LR recombination with R4pMpGWB107.

To construct *pro*Mp*PROTAMINE*:*Lifeact-Venus* (*pro*Mp*PRM*:*LAV*), entry clones containing *LAV* and the *PROTAMINE* promoter were subjected to LR recombination with R4pMpGWB301.

To construct *pro35S*:*GUS*, *pro*Mp*EF1α*:*GUS*, *pro*Mp*HSP17*.*8A1*:*ELuc(PEST)*, *pro*Mp*HSP17*.*8A1*:*mCit-h* and *pro*Mp*HSP17*.*8A1*:*mCit-NLS*, each promoter and entry clone were subjected to LR recombination reaction using the appropriate R4L1pMpGWB destination vectors.

### Generation of transgenic plants

Transformation of *M*. *polymorpha* sporelings was carried out to generate transgenic plants bearing *pro*Mp*EF1α*:*Citrine-PTS1* and *pro35S*:*mRFP1-PTS1*, as described previously [5]. Other transgenic plants in this study were produced using the regenerating thallus transformation method [12]. Transgenic plants were selected using either 0.5 μM 2-chloro-N-[(4-methoxy-6-methyl-1,3,5-triazin-2-yl)aminocarbonyl]-benzenesulfonamide (chlorsulfuron) or 10 mg/l hygromycin as appropriate.

### Confocal microscopy

Plant tissues were examined under an LSM510 META laser scanning confocal microscope equipped with Argon and HeNe lasers (Carl Zeiss, Jena, Germany), as described previously [29]. Emission filters BP535-590 and BP560-615 were used to detect signals from Citrine and mRFP1, respectively.

### Electron microscopy

Thalli were harvested from transgenic plants expressing *Citrine-PTS1* or *mRFP1-PTS1*. Ultrathin sectioning, microscopic analysis and immunogold labelling were performed as previously described [30] with slight modifications. In brief, fixative did not contain 0.06 M sucrose and protein A-gold (AURION, Wageningen, The Netherlands) sizes were 25 nm for catalase and 15 nm for RFP.

### Luciferase assay

Thalli from transgenic plants bearing *pro*Mp*HSP17*.*8A1*:*ELuc(PEST)* were sprayed with 0.8 mM D-luciferin (FUJIFILM Wako Pure Chemical, Osaka, Japan) in 0.01% Triton X-100 and kept in the dark for 15 min. LUC luminescence images were acquired using a high-sensitivity EMCCD camera (ImagEM C9100-13, Hamamatsu Photonics, Hamamatsu, Japan) with a 5 min exposure time and processed with MetaMorph imaging software (Molecular Devices, Tokyo, Japan).

### Histochemical GUS staining

Various organs from transgenic plants bearing *35S* or Mp*EF1α* promoter-*β-glucuronidase* (*GUS*) gene were used for GUS staining using methods [31] with modifications [27] as previously described.

### Nucleotide accession numbers

The nucleotide sequences of the R4pMpGWB and R4L1pMpGWB vectors are registered in DDBJ/GenBank/EMBL under accession numbers AP018588–AP018663.

## Results

### Construction of R4pMpGWB vectors for fusion gene expression under the control of a desired promoter

**Fig 1:**
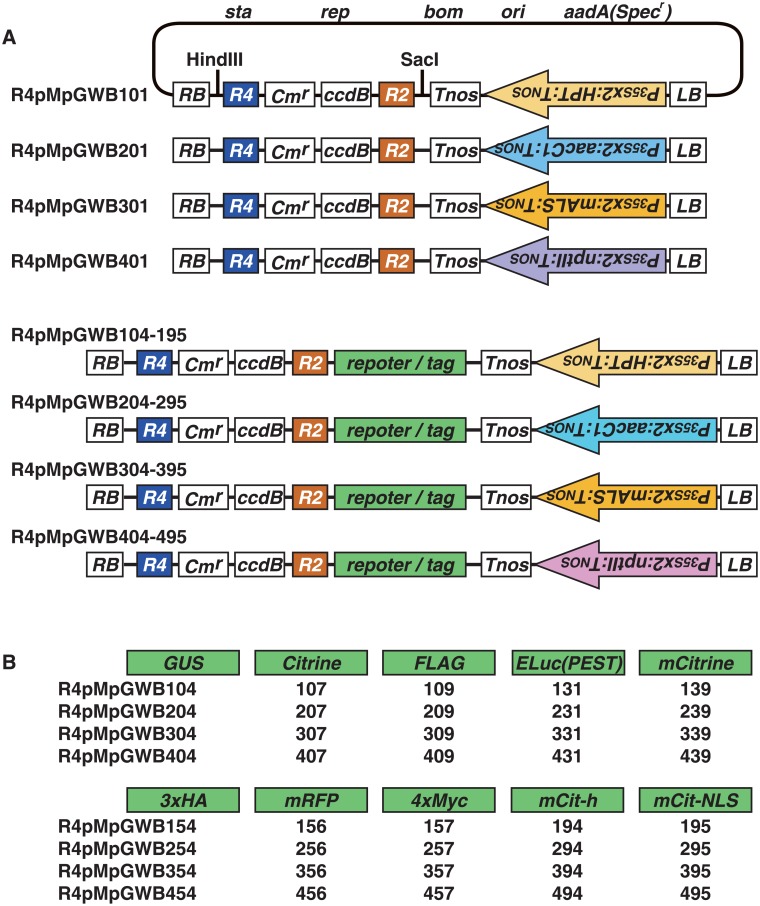
Schematic diagrams of R4pMpGWB vectors. A. Structure of R4pMpGWBs. R4pMpGWB101-195, R4pMpGWB201-295, R4pMpGWB301-395 and R4pMpGWB401-495, containing genes encoding hygromycin phosphotransferase, gentamicin 3′-acetyltransferase, a mutated acetolactate synthase and neomycin phosphotransferase II, respectively, placed in reverse orientation to the Gateway cassette. R4pMpGWB101, R4pMpGWB201, R4pMpGWB301 and R4pMpGWB401 are used for promoter:cDNA constructs, and other vectors are used for promoter:cDNA-reporter/tag fusion constructs. Details of plasmid construction and the vector backbone are given in the Materials and Methods. RB, right border; LB, left border; *R2*, *att*R2; *R4*, *att*R4; *sta*, region conferring stability in *Agrobacterium*; *rep*, broad host-range replication origin; *bom*, *cis*-acting element for conjugational transfer; *ori*, ColE1 replication origin; *aadA*(*Spec*^*r*^), streptomycin/spectinomycin adenyltransferase. *Cm*^*r*^, chloramphenicol resistance marker; *ccd*B, negative selection marker used in bacteria; *P*_*35S*_*x2*, the double-enhancer version of CaMV 35S promoter; *Tnos*, nopaline synthase terminator; *HPT*, hygromycin phosphotransferase; *aacC1*, gentamicin 3′-acetyltransferase; *mALS*, mutated acetolactate synthase; *nptII*, neomycin phosphotransferase II. B. Reporters and tags carried by R4pMpGWBs. GUS, β-glucuronidase; Citrine, improved version of yellow fluorescent protein; FLAG, FLAG-tag; ELuc(PEST), emerald luciferase with PEST sequence; mCitrine, monomeric Citrine; 3×HA, triple HA tag; mRFP, monomeric red fluorescent protein; 4×Myc, four repeats of the Myc tag; mCit-h, mCitrine with ER retention signal; mCit-NLS, mCitrine with nuclear localisation sequence.

Two approaches using pMpGWB vectors [6] as parent materials were used for the generation of R4pMpGWB vectors suitable for the assembly of three DNA fragments for *M*. *polymorpha*. The first strategy was to move the region containing the Gateway cassette and a reporter/tag fragment in the R4pGWB [18] to the corresponding region in pMpGWB vectors [6]. As described in Materials and Methods, intermediate vectors derived from pMpGWB vectors were prepared. Binary vectors containing the genes encoding reporter/tags FLAG, 3×HA, 4×Myc, GUS or mRFP1 were generated using this method. A second strategy was employed for other vectors. The pUGW-based Gateway cloning-compatible vector construction system, which used vectors derived from pUC119 [17–19], was employed as their corresponding pMpGWB vectors were not present. Initially, DNA fragments encoding ELuc(PEST), Citrine, mCitrine, ER-targeted mCitrine (mCit-h) and nucleus-targeted mCitrine (mCit-NLS) were amplified by PCR. The PCR products were subcloned into pUGW vectors to generate intermediate vectors. Then, the responsible regions were digested with appropriate restriction enzymes and ligated with the intermediate vectors from pMpGWB vectors. This generated 44 binary vectors in the R4pMpGWB series, with a hygromycin resistance gene (R4pMpGWB1xx), a gentamicin resistance gene (R4pMpGWB2xx), a mutated acetolactate synthase (mALS) gene for selection with the sulfonylurea herbicide chlorsulfuron (R4pMpGWB3xx) or a kanamycin resistance gene (R4pMpGWB4xx) (Fig 1A) in addition to ten different reporters/tag sequences (Fig 1B). The remaining four vectors (R4pMpGWB101, R4pMpGWB201, R4pMpGWB301 and R4pMpGWB401) did not contain reporters/tags (Fig 1A).

**Fig 2:**
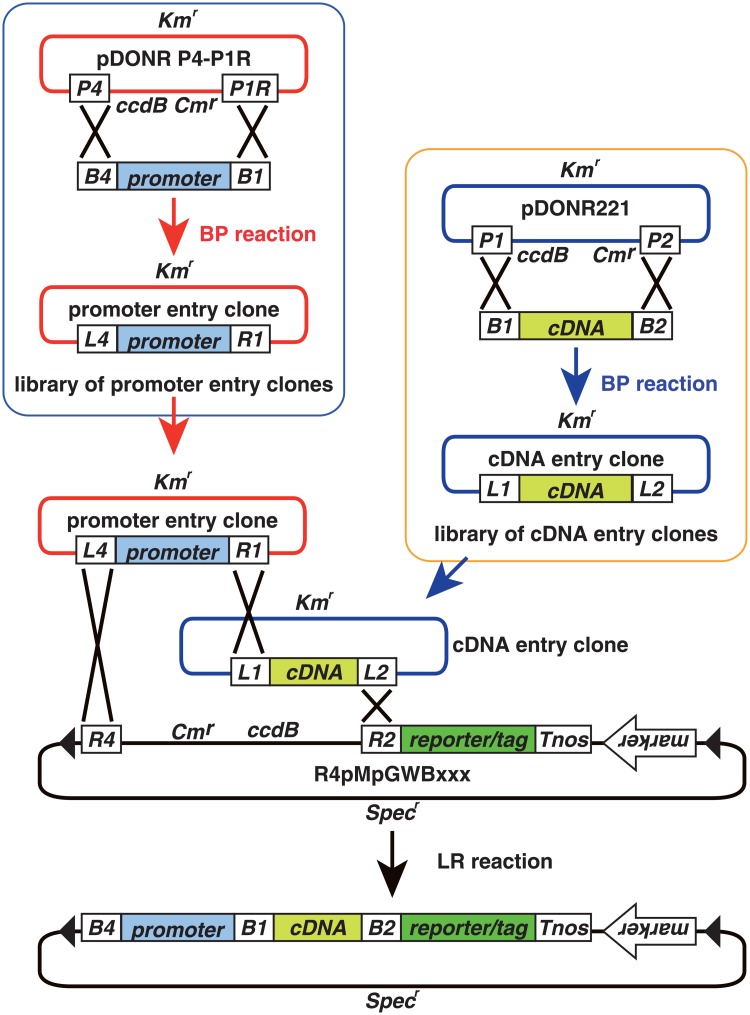
Outline of cloning procedure using the R4pMpGWB system. Promoter entry clones are constructed by a BP reaction between pDONR P4-P1R and an *attB4*-promoter-*attB1* fragment. The cDNA entry clones are constructed by the BP reaction using pDONR221 and the *attB1*-cDNA-*attB2* fragment. The libraries of promoters and cDNAs can be used as resources for construction of chimeric fusions. Promoter and cDNA entry clones and R4pMpGWB vectors are used in a tripartite LR reaction to form a C-terminal fusion of cDNA-encoded protein and a reporter or tag. Note: as pDONR P4-P1R has been discontinued, pENTR 5′-TOPO (Thermo Fisher Scientific) can be used as an alternative for promoter entry clone production. Arrowheads, T-DNA border sequences; *B1*, *att*B*1*; *B2*, *att*B2; *B4*, *att*B4; *P4*, *att*P4; *P1R*, *att*P1R; *L1*, *att*L1; *L2*, *att*L2; *L4*, *att*L4; *R1*, *att*R1; *R2*, *att*R2; *Km*^*r*^, kanamycin-resistant marker; *Cm*^*r*^, chloramphenicol-resistant marker; *Spec*^*r*^, spectinomycin-resistant marker; *ccdB*, negative selection marker used in bacteria.

**Fig 3:**
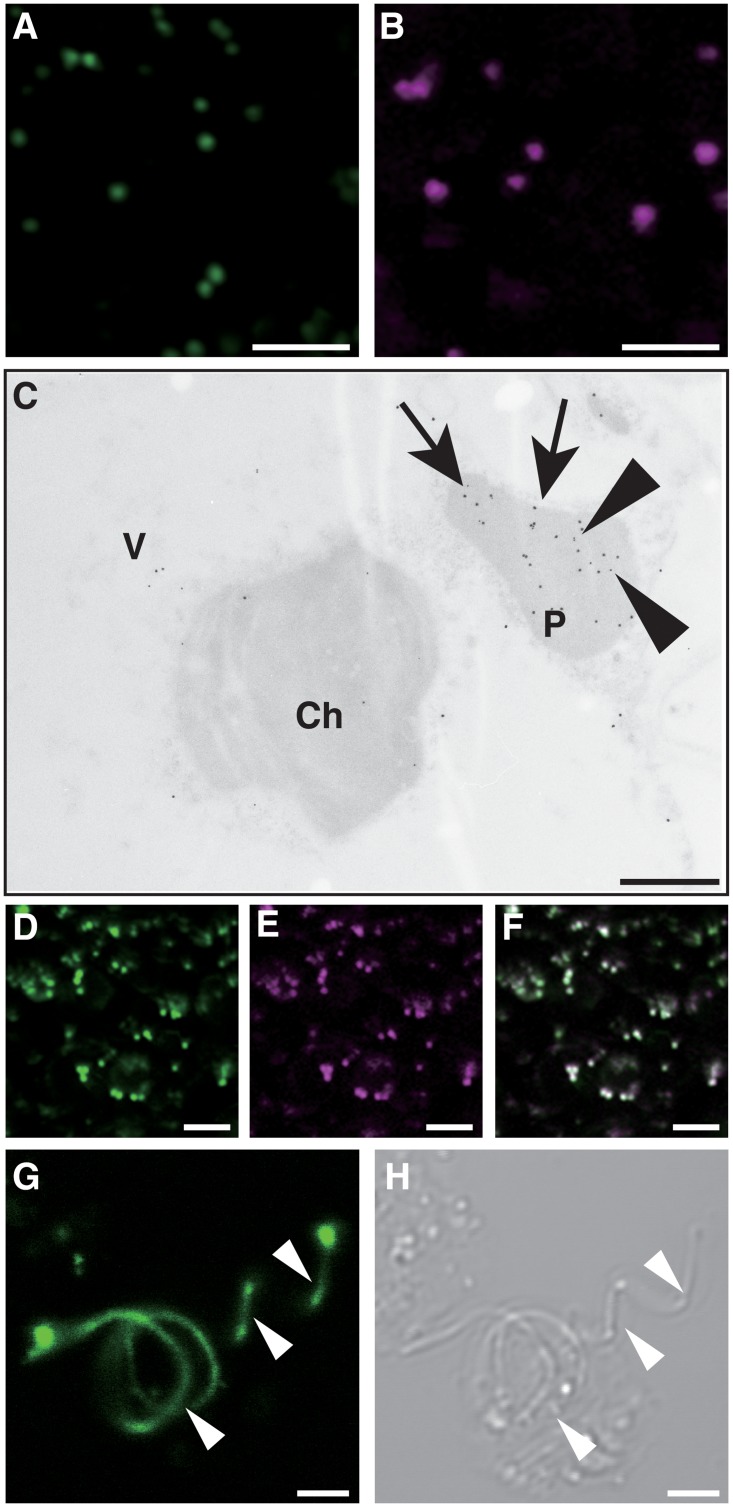
Subcellular localisation of Citrine- and mRFP1-fusion proteins in *M*. *polymorpha*. Thallus epidermal cells and sperm cells in transgenic plants were used for fluorescence observation. A. *pro*Mp*EF1α*:*Citrine-PTS1* transgenic plant. B. *pro35S*:*mRFP1-PTS1* transgenic plant. Bars: 10 μm. C. Immunocytochemical localisation of mRFP1-PTS1 protein. Immunogold labelling of ultrathin sections of thalli in *pro35S*:*mRFP1-PTS1* transgenic plants was carried out using antibodies against RFP (arrowhead) and catalase (arrow). P, peroxisome; Ch, chloroplast; V, vacuole. Bar: 1 μm. D–F. Fluorescence signals from Citrine (D) and mRFP (E) in transgenic plants bearing both *pro35S*:*PTS2-Citrine* and *pro35S*:*mRFP1-PTS1*. (F) represents the merged image of (D) and (E). Bars: 10 μm. G–H. Fluorescence signals from Lifeact-Venus (G) and differential interference contrast image of sperm cells (H). White arrowheads indicate filamentous structures. Bar: 2 μm.

R4pGWB vectors can be used to assemble tripartite DNA fragments by LR recombination reactions and are thus useful for construction of fusions with a promoter, a coding sequence and a reporter/tag (Fig 2). To verify that the R4pMpGWB vectors were capable of compiling tripartite fusions by LR recombination, and that the resultant fusions were functional in *M*. *polymorpha* cells, test constructs for peroxisome visualisation were generated. Initially, transgenic *M*. *polymorpha* plants were generated expressing *Citrine* with peroxisome targeting signal 1 (PTS1) or *mRFP1-PTS1*, constructed from conventional pMpGWB vectors [6]. As shown in Fig 3A and 3B, spherical fluorescence was observed, consistent with observations in transgenic Arabidopsis [29]. Immunoelectron microscopic analysis was used to confirm that these spherical signals represented peroxisomes. Double labelling using antibodies against RFP and against a peroxisome marker, catalase, revealed co-localisation of catalase and mRFP1 (Fig 3C), indicating that the spherical signals were derived from peroxisomes. Next, attempts were made to express *PTS2-Citrine*, which was expected to be imported to peroxisomes via PTS2-dependent protein transport. An R4pMpGWB vector was used to construct the *PTS2-Citrine* fusion gene, and this was transformed into transgenic *M*. *polymorpha* already expressing *mRFP1-PTS1*. Transgenic plants were selected for resistance to the appropriate antibiotics and were shown to contain peroxisomes labelled with both Citrine (Fig 3D) and mRFP1 (Fig 3E), with perfectly merged signals (Fig 3F).

An R4pMpGWB vector was also used to visualise actin filaments in sperm cells. The endogenous *PROTAMINE* promoter (*pro*Mp*PRM*), previously shown to be functional in sperm cells [32], was used alongside *LAV* DNA fragments containing the upstream region of the *PROTAMINE* promoter (-2,740 to +3) were used to generate *pro*Mp*PRM*:*LAV* containing the fusion of the *pro*Mp*PRM* with *LAV* cDNA [26]. Filamentous structures were observed in sperm cells as shown in Fig 3G and 3H.

Together, these results demonstrated that R4pMpGWB series vectors were functional in *M*. *polymorpha* cells.

### Promoter analysis using R4L1pMpGWB vectors

**Fig 4:**
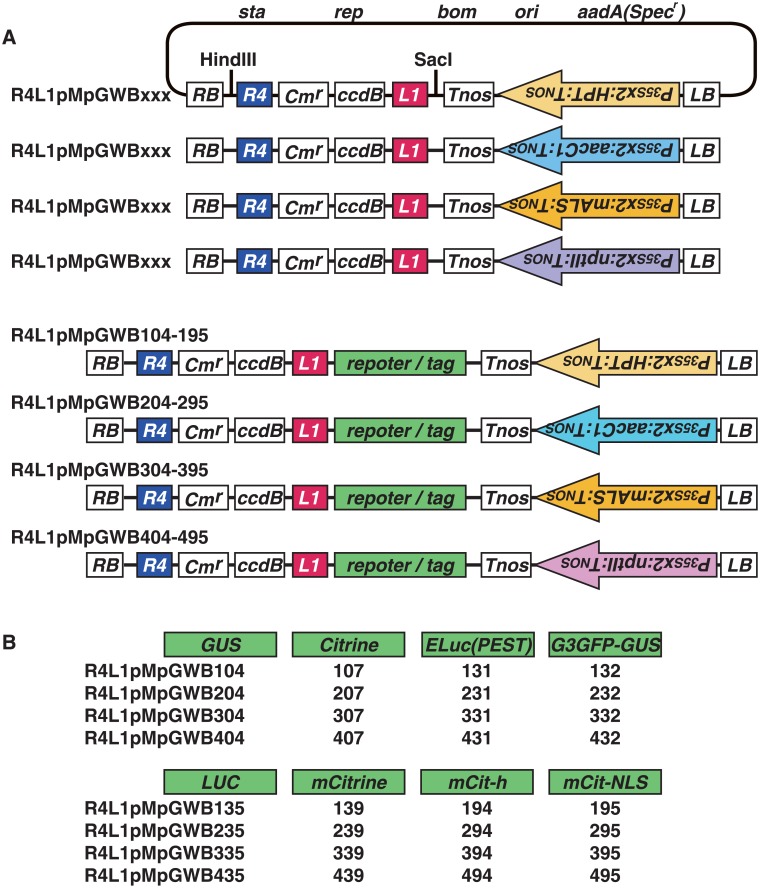
Schematic representations of R4L1pMpGWB vectors. A. R4L1pMpGWB structures. R4L1pMpGWB101-195, R4L1pMpGWB201-295, R4L1pMpGWB301-395 and R4L1pMpGWB401-495 contain genes encoding hygromycin phosphotransferase, gentamicin 3′-acetyltransferase, mutated acetolactate synthase and neomycin phosphotransferase II, respectively, placed in reverse orientation to the Gateway cassette. Details of plasmid construction and the vector backbone are given in the Materials and Methods. RB, right border; LB, left border; *L1*, *att*L1; *R4*, *att*R4; *sta*, region conferring stability in *Agrobacterium*; *rep*, broad host-range replication origin; *bom*, *cis*-acting element for conjugational transfer; *ori*, ColE1 replication origin; *aadA*(*Spec*^*r*^), streptomycin/spectinomycin adenyltransferase. *Cm*^*r*^, chloramphenicol resistance marker; *ccd*B, negative selection marker used in bacteria; *P*_*35S*_*x2*, the double-enhancer version of CaMV 35S promoter; *Tnos*, nopaline synthase terminator; *HPT*, hygromycin phosphotransferase; *aacC1*, gentamicin 3′-acetyltransferase; *mALS*, mutated acetolactate synthase; *nptII*, neomycin phosphotransferase II. B. Reporters included in R4L1pMpGWBs. GUS, β-glucuronidase; Citrine, improved version of yellow fluorescent protein; ELuc(PEST), emerald luciferase with PEST sequence; G3GFP-GUS, G3 green fluorescent protein fused to GUS; Luc, modified luciferase; mCitrine, monomeric Citrine; mCit-h, mCitrine with ER retention signal; mCit-NLS, mCitrine with nuclear localisation sequence.

**Fig 5:**
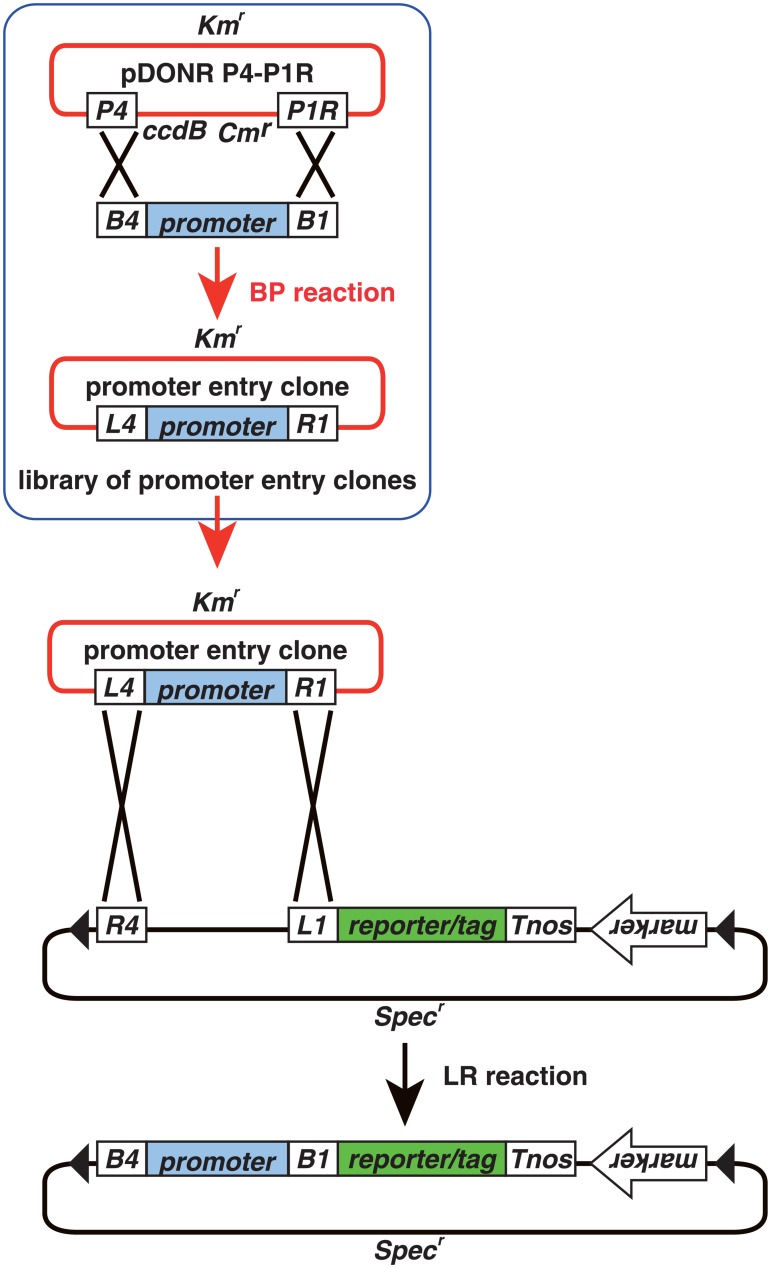
Outline of cloning procedure using the R4pL1MpGWB system. Promoter entry clones are constructed by a BP reaction between pDONR P4-P1R and an *attB4*-promoter-*attB1* fragment. The promoter entry clones and R4L1pMpGWB vectors are used for a bipartite LR reaction. Note: as pDONR P4-P1R has been discontinued, pENTR 5′-TOPO (Thermo Fisher Scientific) can be used as an alternative for promoter entry clone production. Arrowheads, T-DNA border sequences; *B1*, *att*B1; *B4*, *att*B4; *P4*, *att*P4; *P1R*, *att*P1R; *L1*, *att*L1; *L4*, *att*L4; *R1*, *att*R1; *R4*, *att*R4; *Km*^*r*^, kanamycin-resistant marker; *Cm*^*r*^, chloramphenicol-resistant marker; *Spec*^*r*^, spectinomycin-resistant marker; *ccdB*, negative selection marker used in bacteria.

The R4L1pMpGWB vector series was generated for use in promoter analysis. The vector structures were similar to those of R4pMpGWB vectors, except the replacement of *att*R2 with *att*L1 (Fig 4). The promoter entry clones containing *att*L4 and *att*R1, which were generated for the R4pMpGWB vectors, can thus be easily transferred to a region upstream of a reporter sequence in a destination vector without any modifications (Fig 5).

**Fig 6:**
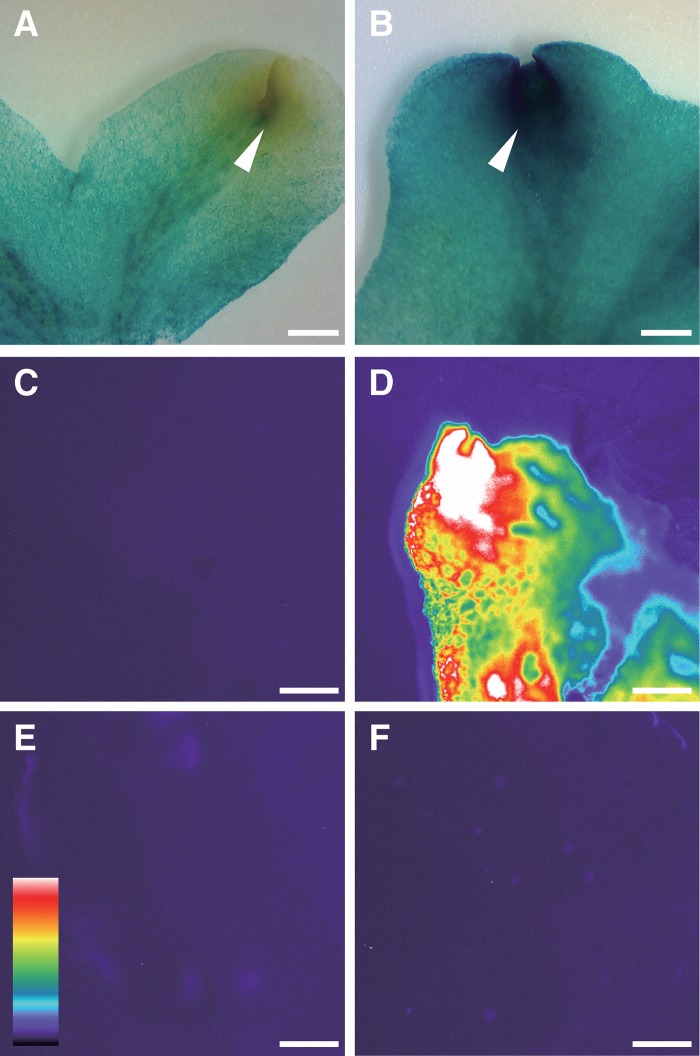
GUS staining and heat-shock analysis of transgenic *M*. *polymorpha* bearing R4L1pMpGWB constructs. (A, B) Thalli of *pro35S*:*GUS* (A) and *pro*Mp*EF1α*:*GUS* (B) transgenic plants were used for GUS staining. Arrowheads indicate meristematic zones. (C–F) Luminescence images of wild-type (C) and transgenic plants bearing *pro*Mp*HSP17*.*8A1*:*ELuc(PEST)* (D–F). Wild-type (C) and transgenic plants (D, E) were treated with heat shock at 37°C for 1 h and then incubated at 22°C for 2 h. (C, D) Images from wild-type (C) and transgenic plants after addition of luciferin. (E) Image from transgenic plant without addition of luciferin. (F) Image from transgenic plant without heat-shock treatment before addition of luciferin. Bars: 1 mm.

To test the utility of the R4L1pMpGWB vectors, expression of *GUS* under the control of the *35S* promoter (Fig 6A) or Mp*EF1α* promoter (Fig 6B) was investigated. Both promoters have roles in constitutive gene expression in various *M*. *polymorpha* cells including cells in the thallus [33]. In the meristematic zone, however, only the Mp*EF1*α promoter, and not the *35S* promoter, drove strong *GUS* gene expression (compare arrows in Fig 6A and 6B). These results were consistent with previous observations [33].

The utility of R4L1pMpGWB vectors was further investigated using a luciferase assay. *Luciferase* was fused to the PEST sequence under the control of the endogenous *HEAT SHOCK PROTEIN17*.*8A1* (Mp*HSP17*.*8A1*) promoter. Expression of Mp*HSP17*.*8A1* was induced by treatment at 37°C for 1 h [34]. In comparison with the wild-type plant (Fig 6C), transgenic plants subjected to heat treatment showed high luminescence levels (Fig 6D). Two transgenic negative controls were used. In the first, the transgenic line was heat treated but the luciferin substrate was not added (Fig 6E). In the second, luciferin was added but no heat induction was applied (Fig 6F). As expected, no luminescence was observed in these control plants (Fig 6E and 6F).

**Fig 7:**
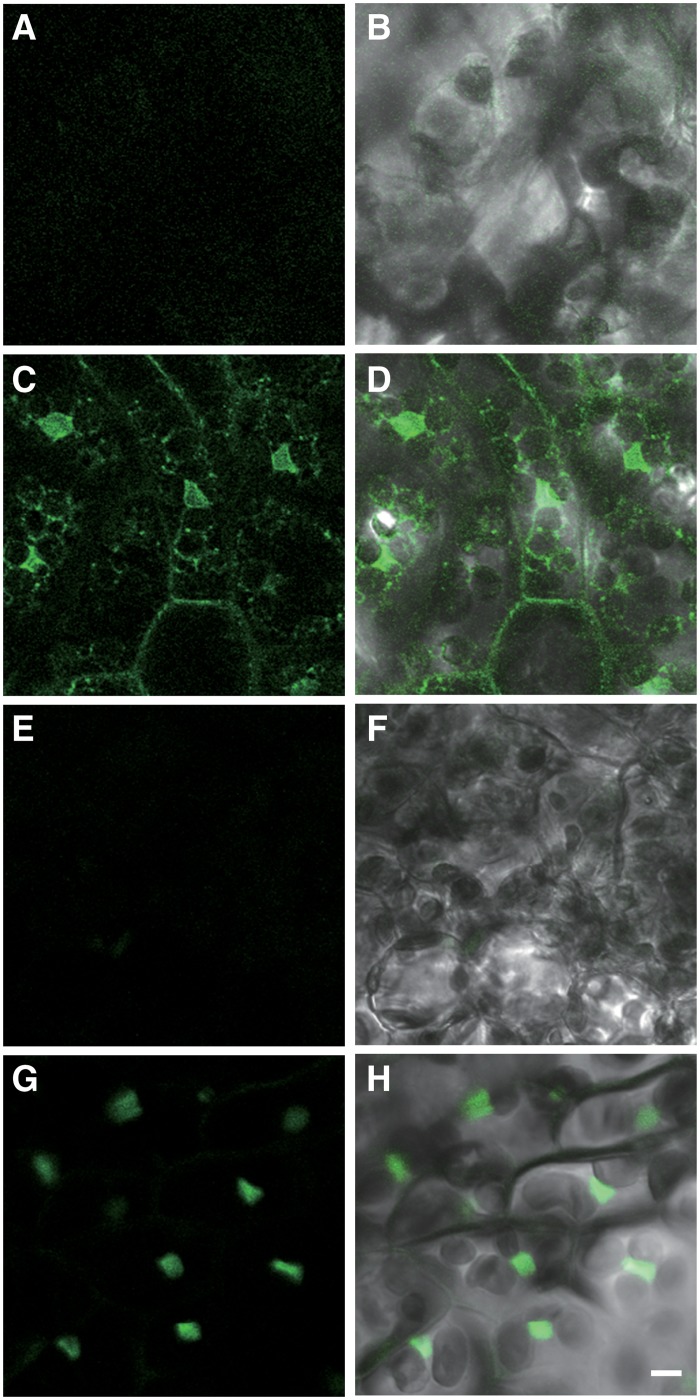
Observation of ER- or nuclear-targeted Citrine in transgenic *M*. *polymorpha*. (A–H) Heat-shock treatment was performed on transgenic plants bearing *pro*Mp*HSP17*.*8A1*:*mCit-h* (A–D) and *pro*Mp*HSP17*.*8A1*:*mCit-NLS* (E–H) as described by Nishihama et al. (2016) [34]. Fluorescent microscopic observations of Citrine fluorescence were performed before (A, B, E and F) and after (C, D, G and H) heat-shock treatment. (B, D, F and H) show the merged images of Citrine signals with differential interference contrast images. Bar: 10 μm.

Finally, R4L1pMpGWB vectors were generated with organelle-localised mCitrine as a reporter. Targeting to the ER and nucleus was selected. ER- or nucleus-targeted mCitrine sequence was fused to the Mp*HSP17*.*8A1* promoter and expressed in *M*. *polymorpha*. As shown in Fig 7, fluorescence signals were observed in cells after heat treatment (Fig 7C, 7D, 7G and 7H), but not before heat treatment (Fig 7A, 7B, 7E and 7F). ER-targeted mCitrine showed interconnected network-like structures and flattened sacs (Fig 7C and 7D). In the cells expressing nucleus-targeted mCitrine, fluorescence was observed as one large structure in each cell (Fig 7G and 7H). The fluorescence patterns seen in transgenic lines were consistent with those in previous reports in *M*. *polymorpha* [35] and Arabidopsis using GFP [36, 37].

Overall, our results showed that the R4L1pMpGWB vectors functioned well in *M*. *polymorpha* cells and were ideal for vector construction for promoter analysis.

## Discussion

In this study, we demonstrated two efficient construction systems for the generation of fusion genes for *M*. *polymorpha*. Many vectors have been developed for generating transgenic plants in model systems such as tobacco and Arabidopsis, some of which may be applicable to *M*. *polymorpha*. However, the newly developed R4pMpGWB and R4L1pMpGWB vectors are compatible with Gateway cloning technology, facilitating the rapid production of a range of fusion constructs without the need for laborious conventional methods involving digestion with restriction enzymes, ligation and subcloning. Using these novel vectors, we were able to generate some transgenic *M*. *polymorpha* simultaneously. As described above, the fusions of promoter:cDNA—reporter/tag and promoter:cDNA were constructed efficiently and rapidly, and worked in *M*. *polymorpha* cells as expected, thus the detection of organelle-dependent fluorescent signals (Figs 3D–3G and 7), and the promoter-specific expression such as sperm cell-specific visualization of actin filaments (Fig 3D) and heat-inducible gene expression (Figs 6 and 7). These results show that the construction systems using R4pMpGWB and R4L1pMpGWB vectors are powerful tools to efficiently generate transgenic plants. In addition, both vector series enable the use of four resistance genes (hygromycin, gentamicin, chlorsulfuron and G418), extending the range of choices to facilitate re-transformation of pre-existing transgenic *M*. *polymorpha* lines (Fig 3D–3F).

### Features of the R4pMpGWB and R4L1pMpGWB vector series

The recombination reaction for R4pMpGWB vectors requires *att*L1 and *att*L2 sites in the entry clone with the coding sequence (Fig 2). Most users of Gateway cloning technology generate entry clones containing *att*L1 and *att*L2 sites, and such entry clones can thus be generated for the R4pMpGWB vectors without the need for any *att* sequence modifications. The *att*L4 and *att*R1 sites in a promoter entry clone are required for recombination with the *att*R4 site in the vector and the *att*L1 site in an entry containing a coding sequence (Fig 2). This type of promoter entry clone is also compatible with R4L1pMpGWB vectors as the R4L1pMpGWB vectors contain both *att*R4 and *att*L1 sites (Fig 5). Thus, the R4pMpGWB and R4L1pMpGWB vectors can use promoter entry clones that have already been generated. To date, promoter entry clones have been produced by BP reaction between PCR products with *att* sequences and pDONR P4-P1R (Thermo Fisher Scientific). However, as pDONR P4-P1R has been discontinued, pENTR 5′-TOPO (Thermo Fisher Scientific) can be used as an alternative for promoter entry clone production.

R4pMpGWB vectors encode a reporter or tag fused to the C-terminus of a protein or a peptide derived from an entry clone with a desired coding sequence (Fig 2). N-terminal subcellular targeting tags can be used with R4pMpGWB vectors without modification. For example, PTS2 functions only when placed ahead of a reporter, at the N-terminal end of a protein [28, 38]. Improper fusion of PTS2, such as fusion to the C-terminal end, eliminates targeting to peroxisomes. Here, the *pro35S*:*PTS2-Citrine* fusion was easily generated by LR recombination between pDONR35SproDup, pPTS2-221 and R4pMpGWB107 (Fig 3D, S2 Table). Where a C-terminal fusion is required, an alternative method is needed to use LR recombination. One method is to prepare DNA fragments containing both a coding sequence and a reporter/tag sequence as this entry clone. This entry clone can then be used with LR recombination between a promoter entry clone and one of the R4pMpGWB vectors without reporters/tags (R4pMpGWB101, R4pMpGWB201, R4pMpGWB301 and R4pMpGWB401).

Ten different reporters or tags are available for R4pMpGWB vectors (Fig 1) but, as the R4L1pMpGWB vectors are used for promoter assays, only GUS staining and fluorescent or bioluminescent imaging reporters are available for R4L1pMpGWB vectors (Fig 4). Two DNA fragments are assembled for R4L1pMpGWB vectors, and this is therefore a more efficient LR reaction than the assembly of three fragments in the R4pMpGWB system.

Organelle-targeted mCitrine was included in the reporter repertoire for both vector series in response to demand from researchers. The pMpGWB vectors containing nucleus-localised Citrine and tdTomato were constructed previously by Ishizaki et al. (2015) [6] and used for visualisation of transformed cells. Here, mCitrine was used rather than Citrine and tdTomato and was found to give similar performance (Fig 7G and 7H). In addition, vectors were generated containing ER-targeted mCitrine. ER is the largest subcellular compartment and is distributed throughout the cell as tubular and/or network-like structures (Fig 7C and 7D). It is therefore expected that visualisation of cells transformed with ER-targeted mCitrine will be easier than visualisation of nucleus-localised cells.

The mRFP1-based fusion functioned well in *M*. *polymorpha* cells (Fig 3). Alternative red fluorescent protein fusions available in the pMpGWB vector series are TagRFP and tdTomato [6]. The wavelengths of excitation maxima of TagRFP and tdTomato are 554 and 555 nm, respectively, and the wavelengths of emission maxima are 581 and 584 nm, respectively. The wavelengths of excitation and emission maxima of mRFP1 are 584 and 607 nm, respectively, representing an approximately 30 nm red shift compared with TagRFP and tdTomato. This facilitates discrimination between Citrine and RFP signals in co-localisation analysis.

The R4pGWB and R4L1pGWB vector series [18, 20] have been used to generate transgenic Arabidopsis by many plant researchers. Of many ecotypes in Arabidopsis, ecotype Columbia is frequently used as a model material, in which both R4pGWB and R4L1pGWB vectors are known to work well. However, it is not reported whether both vectors can drive resistance gene expression in other Arabidopsis ecotypes. When the R4pGWB and R4L1pGWB vector series are not suitable for generating transgenic plants using other Arabidopsis ecotypes, novel R4pMpGWB and R4L1pMpGWB vectors can be expected to become one of the alternative options to generate transgenic plants.

As new reporters and tags become available, such as fluorescent proteins with improved fluorescence intensity and different wavelengths of excitation and emission, Gateway cloning-compatible vectors for *M*. *polymorpha* will be generated by incorporating these new reporters/tags into the R4pMpGWB and R4L1pMpGWB vector series. These new vector series will be expected to function in other plants, such as soybean and rice, in addition to *M*. *polymorpha*, expecting generation of various transgenic plants efficiently.

### Protein transport to peroxisomes in *M*. *polymorpha*

Previously, we examined the molecular mechanisms of plant peroxisome dynamics by identification of factors for protein transport, division and quality control of peroxisomes in Arabidopsis [27, 29, 39–41]. Similar studies were performed in *M*. *polymorpha* to determine whether peroxisome dynamics were conserved among plant species or were species-specific. Transgenic *M*. *polymorpha* expressing *Citrine-PTS1*, *mRFP1-PTS1* and *PTS2-Citrine* were generated (Fig 3A–3F). The results from these transgenic *M*. *polymorpha* clearly showed that morphology, size and movement of peroxisomes in *M*. *polymorpha* strongly resembled those in Arabidopsis [29], and that PTS1- and PTS2-dependent protein transport pathways were functional in *M*. *polymorpha*. Bioinformatic analysis of 21 peroxin (PEX) sequences in Arabidopsis [42] identified 17 candidate orthologs in *M*. *polymorpha* (unpublished results). Although Arabidopsis PEX3, PEX11 and PEX19 constitute a protein family, the *M*. *polymorpha* orthologs are essentially produced by a single gene. The proteins include PEX5 and PEX7, which are the receptors for PTS1 and PTS2, respectively. These results are consistent with the observations in this study showing that Citrine and mRFP1 were transported to peroxisomes via PTS1- and PTS2-dependent protein transport pathways. Our next approach will be to generate transgenic plants to analyse the function of the peroxisomal candidate proteins in *M*. *polymorpha*. The vector series generated in this study will be valuable tools for plant peroxisome research as well as for other research in *M*. *polymorpha*.

## Supporting information

S1 Table Primer sequences. (DOCX)

S2 Table Entry clones and destination vectors for LR recombination. (DOCX)

## References

[1] Yamato KT, Ishizaki K, Fujisawa M, Okada S, Nakayama S, Fujishita M, et al Gene organization of the liverwort Y chromosome reveals distinct sex chromosome evolution in a haploid system. Proc Natl Acad Sci USA. 2007; 104(15):6472–7. 10.1073/pnas.0609054104 17395720 PMC1851093

[2] Bowman JL, Kohchi T, Yamato KT, Jenkins J, Shu S, Ishizaki K, et al Insights into land plant evolution garnered from the *Marchantia polymorpha* genome. Cell. 2017:287–304. 10.1016/j.cell.2017.09.030 28985561

[3] Ohyama K, Fukuzawa H, Kohchi T, Shirai H, Sano T, Sano S, et al Chloroplast gene organization deduced from complete sequence of liverwort *Marchantia polymorpha* chloroplast DNA. Nature. 1986; 322:572–4.

[4] Oda K, Yamato K, Ohta E, Nakamura Y, Takemura M, Nozato N, et al Gene organization deduced from the complete sequence of liverwort *Marchantia polymorpha* mitochondrial DNA: A primitive form of plant mitochondrial genome. J Mol Biol. 1992; 223(1):1–7. 1731062 10.1016/0022-2836(92)90708-r

[5] Ishizaki K, Chiyoda S, Yamato KT, Kohchi T. *Agrobacterium*-mediated transformation of the haploid liverwort *Marchantiapolymorpha* L., an emerging model for plant biology. Plant Cell Physiol. 2008; 49(7):1084–91. 10.1093/pcp/pcn085 18535011

[6] Ishizaki K, Nishihama R, Ueda M, Inoue K, Ishida S, Nishimura Y, et al Development of Gateway binary vector series with four different selection markers for the liverwort *Marchantia polymorpha*. PLoS One. 2015; 10(9):e0138876 10.1371/journal.pone.0138876 26406247 PMC4583185

[7] Ishizaki K, Nishihama R, Yamato KT, Kohchi T. Molecular genetic tools and techniques for *Marchantia polymorpha* research. Plant Cell Physiol. 2016; 57(2):262–70. 10.1093/pcp/pcv097 26116421

[8] Tanaka D, Ishizaki K, Kohchi T, Yamato KT. Cryopreservation of gemmae from the liverwort *Marchantia polymorpha* L. Plant Cell Physiol. 2016; 57(2):300–6. 10.1093/pcp/pcv173 26561534 PMC4788409

[9] Takenaka M, Yamaoka S, Hanajiri T, Shimizu-Ueda Y, Yamato KT, Fukuzawa H, et al Direct transformation and plant regeneration of the haploid liverwort *Marchantia polymorpha* L. Transgenic Res. 2000; 9:179–85. 11032366 10.1023/a:1008963410465

[10] Chiyoda S, Linley PJ, Yamato KT, Fukuzawa H, Yokota A, Kohchi T. Simple and efficient plastid transformation system for the liverwort *Marchantia polymorpha* L. suspension-culture cells. Transgenic Res. 2007; 16:41–9. 10.1007/s11248-006-9027-1 17103028

[11] Chiyoda S, Ishizaki K, Kataoka H, Yamato KT, Kohchi T. Direct transformation of the liverwort *Marchantia polymorpha* L. by particle bombardment using immature thalli developing from spores. Plant Cell Rep. 2008; 27:1467–73. 10.1007/s00299-008-0570-5 18553085

[12] Kubota A, Ishizaki K, Hosaka M, Kohchi T. Efficient *agrobacterium*-mediated transformation of the liverwort *Marchantia polymorpha* using regenerating Thalli. Biosci Biotechnol Biochem. 2013; 77(1):167–72. 10.1271/bbb.120700 23291762

[13] Tsuboyama S, Kodama Y. AgarTrap: A simplified *agrobacterium*-mediated transformation method for sporelings of the liverwort *Marchantia polymorpha* L. Plant Cell Physiol. 2013; 55(1):229–36. 10.1093/pcp/pct168 24259681

[14] Chiyoda S, Yamato KT, Kohchi T. Plastid transformation of sporelings and suspension-cultured cells from the liverwort *Marchantia polymorpha* L. Methods Mol Biol. 2014; 1132:439–7. 10.1007/978-1-62703-995-6_30 24599873

[15] Tsuboyama-Tanaka S, Kodama Y. AgarTrap‐mediated genetic transformation using intact gemmae/gemmalings of the liverwort *Marchantia polymorpha* L. J Plant Res. 2015; 128:337–44. 10.1007/s10265-014-0695-2 25663453

[16] Bowman JL, Araki T, Arteaga-Vazquez MA, Berger F, Dolan L, Haseloff J, et al The naming of names: Guidelines for gene nomenclature in *Marchantia*. Plant Cell Physiol. 2016; 57(2):257–61. 10.1093/pcp/pcv193 26644462 PMC4788412

[17] Nakagawa T, Suzuki T, Murata S, Nakamura S, Hino T, Maeo K, et al Improved gateway binary vectors: high-performance vectors for creation of fusion constructs in transgenic analysis of plants. Biosci Biotechnol Biochem. 2007; 71:2095–100. 10.1271/bbb.70216 17690442

[18] Nakagawa T, Nakamura S, Tanaka K, Kawamukai M, Suzuki T, Nakamura K, et al Development of R4 Gateway binary bectors (R4pGWB) enabling high-throughput promoter swapping for plant research. Biosci Biotechnol Biochem. 2008; 72:624–9. 10.1271/bbb.70678 18256458

[19] Hino T, Tanaka Y, Kawamukai M, Nishimura K, Mano S, Nakagawa T. Two Sec13p homologs, AtSec13A and AtSec13B, redundantly contribute to formation of COPII transport vesicles in *Arabidopsis thaliana*. Biosci Biotechnol Biochem. 2011; 75:1848–52. 10.1271/bbb.110331 21897010

[20] Tanaka Y, Shibahara K, Nakagawa T. Development of Gateway binary bectors R4L1pGWB possessing the bialaphos resistance gene (bar) and the tunicamycin resistance gene as markers for promoter analysis in plants. Biosci Biotechnol Biochem. 2013; 77(8):1795–7. 10.1271/bbb.130405 23924715

[21] Kamigaki A, Nito K, Hikino K, Goto-Yamada S, Nishimura M, Nakagawa T, et al Gateway bectors for simultaneous detection of multiple protein−protein interactions in plant cells using bimolecular fluorescence complementation. PLoS One. 2016; 11(8):e0160717 10.1371/journal.pone.0160717 27490375 PMC4973907

[22] Aboulela M, Tanaka Y, Nishimura K, Mano S, Nishimura M, Ishiguro S, et al Development of an R4 dual-site (R4DS) gateway cloning system enabling the efficient simultaneous cloning of two desired sets of promoters and open reading frames in a binary vector for plant research. PLoS One. 2017; 12(5):e0177889 10.1371/journal.pone.0177889 28520787 PMC5433782

[23] Aboulela M, Tanaka Y, Nishimura K, Mano S, Kimura T, Nakagawa T. A dual-site gateway cloning system for simultaneous cloning of two genes for plant transformation. Plasmid. 2017; 92:1–11. 10.1016/j.plasmid.2017.05.001 28499723

[24] Gamborg OL, Miller RA, Ojima K. Nutrient requirements of suspension cultures of soybean root cells. Exp Cell Res. 1968; 50(1):151–8. 5650857 10.1016/0014-4827(68)90403-5

[25] Nakamura S, Nakao A, Kawamukai M, Kimura T, Ishiguro S, Nakagawa T. Development of gateway binary vectors, R4L1pGWBs, for promoter analysis in higher plants. Biosci Biotechnol Biochem. 2009; 73(11):2556–9. 10.1271/bbb.90720 19897887

[26] Era A, Tominaga M, Ebine K, Awai C, Saito C, Ishizaki K, et al Application of Lifeact reveals F-actin dynamics in *Arabidopsis thaliana* and the liverwort, *Marchantia polymorpha*. Plant Cell Physiol. 2009; 50(6):1041–8. 10.1093/pcp/pcp055 19369273 PMC2694730

[27] Mano S, Nakamori C, Fukao Y, Araki M, Matsuda A, Kondo M, et al A defect of peroxisomal membrane protein 38 causes enlargement of peroxisomes. Plant Cell Physiol. 2011; 52(12):2157–72. 10.1093/pcp/pcr147 22034551

[28] Kato A, Hayashi M, Mori H, Nishimura M. Molecular characterization of a glyoxysomal citrate synthase that is synthesized as a precursor of higher molecular mass in pumpkin. Plant Mol Biol. 1995; 27:377–90. 7888626 10.1007/BF00020191

[29] Mano S, Nakamori C, Hayashi M, Kato A, Kondo M, Nishimura M. Distribution and characterization of peroxisomes in Arabidopsis by visualization with GFP: Dynamic morphology and actin-dependent movement. Plant Cell Physiol. 2002; 43:331–41. 11917088 10.1093/pcp/pcf037

[30] Hayashi M, Toriyama K, Kondo M, Nishimura M. 2,4-dichlorophenoxybutyric acid-resistant mutants of Arabidopsis have defects in glyoxysomal fatty acid β-oxidation. Plant Cell. 1998; 10:183–95. 9490742 10.1105/tpc.10.2.183 PMC143991

[31] Jefferson RA, Kavanagh TA, Bevan MW. GUS fusions: β-glucuronidase as a sensitive and versatile gene fusion marker in higher plants. EMBO J. 1987; 6:3901–7. 3327686 10.1002/j.1460-2075.1987.tb02730.x PMC553867

[32] Higo A, Niwa M, Yamato KT, Yamada L, Sawada H, Sakamoto T, et al Transcriptional framework of male gametogenesis in the liverwort *Marchantia polymorpha* L. Plant Cell Physiol. 2016; 57(2):325–38. 10.1093/pcp/pcw005 26858289

[33] Althoff F, Kopischke S, Zobell O, Ide K, Ishizaki K, Kohchi T, et al Comparison of the *MpEF1a* and *CaMV35* promoters for application in *Marchantia polymorpha* overexpression studies. Transgenic Res. 2014; 23:235–44. 10.1007/s11248-013-9746-z 24036909

[34] Nishihama R, Ishida S, Urawa H, Kamei Y, Kohchi T. Conditional gene expression/deletion systems for *Marchantia polymorpha* using its own heat-shock promoter and Cre/loxP-mediated site-specific recombination. Plant Cell Physiol. 2016; 57(2):271–80. 10.1093/pcp/pcv102 26148498

[35] Ogasawara Y, Ishizaki K, Kohchi T, Kodama Y. Cold-induced organelle relocation in the liverwort *Marchantia polymorpha* L. Plant, Cell Environ. 2013; 36:1520–8. 23421791 10.1111/pce.12085

[36] Chytilova E, Macas J, Galbraith DW. Green fluorescent protein targeted to the nucleus, a transgenic phenotype useful for studies in plant biology. Annals Bot. 1999; 83:645–54.

[37] Ridge RW, Uozumi Y, Plazinski J, Hurley UA, Williamson RE. Developmental transitions and dynamics of the cortical ER of *Arabidopsis* cells seen with green fluorescent protein. Plant Cell Physiol. 1999; 40(12):1253–61. 10682347 10.1093/oxfordjournals.pcp.a029513

[38] Kato A, Hayashi M, Kondo M, Nishimura M. Targeting and processing of a chimeric protein with the N-terminal presequence of the precursor to glyoxysomal citrate synthase. Plant Cell. 1996; 8:1601–11. 10.1105/tpc.8.9.1601 8837511 PMC161301

[39] Mano S, Nakamori C, Nito K, Kondo M, Nishimura M. The Arabidopsis *pex12* and *pex13* mutants are defective in both PTS1- and PTS2-dependent protein transport to peroxisomes. Plant J. 2006; 47:604–18. 10.1111/j.1365-313X.2006.02809.x 16813573

[40] Goto S, Mano S, Nakamori C, Nishimura M. *Arabidopsis* ABERRANT PEROXISOME MORPHOLOGY9 is a peroxin that recruits the PEX1-PEX6 complex to peroxisomes. Plant Cell. 2011; 23:1573–87. 10.1105/tpc.110.080770 21487094 PMC3101541

[41] Goto-Yamada S, Mano S, Yamada K, Oikawa K, Hosokawa Y, Hara-Nishimura I, et al Dynamics of the light-dependent transition of plant peroxisomes. Plant Cell Physiol. 2015; 56(7):1264–71. 10.1093/pcp/pcv081 26063394

[42] Kamada T, Nito K, Hayashi H, Mano S, Hayashi M, Nishimura M. Functional differentiation of peroxisomes revealed by expression profiles of peroxisomal genes in *Arabidopsis thaliana*. Plant Cell Physiol. 2003; 44:1275–89. 14701923 10.1093/pcp/pcg173

