# Supplementary material for: Novel gateway binary vectors for rapid tripartite DNA assembly and promoter analysis with various reporters and tags in the liverwort Marchantia polymorpha
Source: PLoS One. 2018 Oct 4;13(10):e0204964. doi: 10.1371/journal.pone.0204964 (PMC6171868; doi:10.1371/journal.pone.0204964)
Supplement: S1 Table — (DOCX) [file pone.0204964.s001.docx]

| **S1 Table. Primer sequences used in this study** | |
| --- | --- |
| **Primer name** | **Nucleotide sequence** |
| Citrine-F | 5'-ATGGTGAGCAAGGGCGAGG-3' |
| Citrine-R | 5'-CTTGTACAGCTCGTCCATGC-3' |
| ElucP-F | 5'-ATGGAGAGAGAGAAGAACGTG-3' |
| ElucP-R | 5'-CTACACATTGATCCTAGCAGA-3' |
| AtcCit-F | 5'-ATGGTGTCTAAGGGTGAGGA-3' |
| AtcCit-R | 5'-CTTGTAAAGCTCATCCATTCC-3' |
| ElucP-sdF | 5'-AGAAGGCTTCTAAGCTGAGCCATGGCTTCCCGC -3' |
| ElucP-sdR | 5'-GCGGGAAGCCATGGCTCAGCTTAGAAGCCTTCT -3' |
| AtcCitNLSv2-R1 | 5'-TCTTCTTCTTAGGCTGCTTGTAAAGCTCATCCATTC -3' |
| AtcCitNLSv2-R2 | 5'-CTATCCTCCAACCTTTCTCTTCTTCTTAGGCTG -3' |
| mCit-hF1 | 5'-CTACCGCACCATGGGCGGCATGGTGTCTAAGGGTGAG -3' |
| mCit-hR1 | 5'-CCCCCGAGATCTCCCTTGTAAAGCTCATCCATTC -3' |
| mCit-hF2 | 5'-AGTGGCTCTGCTGGTTGCAGATGCGTACGCCTACCGCACCATGGG -3' |
| mCit-hR2 | 5'-GGTGGTGGTGGTGGTGCCCCCCCCCGAGATCTCC -3' |
| mCit-hF3 | 5'-ATGGCCAGACTCACAAGCATCATTGCCCTCTTCGCAGTGGCTCTGCTGGT -3' |
| mCit-hR3 | 5'-TCAAAGCTCATCGTGGTGGTGGTGGTGGT -3' |
| 35SpDup-FB4 | 5'-ATAGAAAAGTTGTTTGGCTAGAGCAGCTTGCCAAC -3' |
| 35SpDup-RB1R | 5'-TTTGTACAAACTTGCAGAGATAGATTTGTAGAGAGAGAC -3' |
| EF1pro-FB4 | 5'-ATAGAAAAGTTGTTCAAATGAGTCACACACATTGTT -3' |
| EF1pro-RB1R | 5'-TTTGTACAAACTTGCCAACCTTTCTGCAGGCACA -3' |
| MpHSP17.8A1pro-FB4 | 5'- ATAGAAAAGTTGTTCCAGCCATTATAGCAACTATG -3' |
| MpHSP17.8A1pro-RB1R | 5'- TTTGTACAAACTTGCGGTGGAACTTCGCTCTCGA -3' |
| MpPRMproF3-B4 | 5'- ATAGAAAAGTTGTTCGTTTTGAGGGTTCCTCTGT -3' |
| MpPRMproR-RB1Rv2 | 5'- TTTGTACAAACTTGCCATGCTTGAAACTCTCCCCTTC -3' |
| Venus-FB1 | 5'-AAAAAGCAGGCTTTATGGTGAGCAAGGGCGAGG -3' |
| EF1pro-RB1R | 5'-AGAAAGCTGGGTTTCATAGCTTCGAAAC CTTGTACAGCTCGTCCAT -3' |
| LAV-FB1 | 5'-AAAAAGCAGGCTTTATGGGTGTCGCAGATTTGATC -3' |
| LAV-RB2 | 5'-AGAAAGCTGGGTTTTACTTGTACAGCTCGTCCAT -3' |
