# Supplementary material for: Novel gateway binary vectors for rapid tripartite DNA assembly and promoter analysis with various reporters and tags in the liverwort Marchantia polymorpha
Source: PLoS One. 2018 Oct 4;13(10):e0204964. doi: 10.1371/journal.pone.0204964 (PMC6171868; doi:10.1371/journal.pone.0204964)
Supplement: S2 Table — (DOCX) [file pone.0204964.s002.docx]

| **S2 Table. Entry clones and destination vectors for LR recombination reaction to generate fusion genes in this study** | | | | |
| --- | --- | --- | --- | --- |
| **Fusion gene** | **Entry clone (Promoter)** | **Entry clone (cDNA)** | **Destination vector** | **Use in this study** |
| *pro*Mp*EF1α:Citrne-PTS1* | - | pDONRCitrine-PTS1 | pMpGWB303 | Visualization of peroxisome with Citrine |
| *pro35S:mRFP1-PTS1* | - | pDONRmRFP-PTS1^*^ | pMpGWB302 | Visualization of peroxisome with mRFP1 |
| *pro35S:PTS2-Citrine* | pDONR35SproDup | pPTS2-221^*^ | R4pMpGWB107 | Visualization of peroxisome with Citrine and mRFP1 |
| *pro*Mp*PRM:LAV* | pDONRMpPRMproS | pDONRLAV | R4pMpGWB301 | Visualization of actin filament with Lifeact-Venus |
| *pro35S:GUS* | pDONR35SproDup | - | R4L1pMpGWB304 | GUS staining |
| *pro*Mp*EF1α:GUS* | pDONREF1pro | - | R4L1pMpGWB304 | GUS staining |
| *pro*Mp*HSP17.8A1:Eluc(PEST)* | pDONRMpHSP17.8pro | - | R4L1pMpGWB331 | Luminescence observation by heat shock treatment |
| *pro*Mp*HSP17.8A1:mCit-h* | pDONRMpHSP17.8pro | - | R4L1pMpGWB394 | Visualization of ER by heat shock treatment |
| *pro*Mp*HSP17.8A1:mCit-NLS* | pDONRMpHSP17.8pro | - | R4L1pMpGWB395 | Visualization of nucleus by heat shock treatment |
| ^*^This entry clone was described previously [39]. | | | | |
